# Supplementary material for: Neural correlates of the uncanny valley effect for robots and hyper-realistic masks
Source: PLoS One. 2025 Mar 18;20(3):e0311714. doi: 10.1371/journal.pone.0311714 (PMC11957730; doi:10.1371/journal.pone.0311714)
Supplement: Attachment — Submitted filename: Peer Review 3 OSQ.pdf [file pone.0311714.s001.pdf]

Peer Review

"Neural Correlates of the Uncanny Valley Effect for Robots and Hyper-Realistic Masks"

❖ *Summary of the Manuscript*

Title: *"Neural Correlates of the Uncanny Valley Effect for Robots and Hyper-Realistic Masks"*

Objective: *This paper takes on the intriguing challenge of exploring neural responses linked to the uncanny valley effect. By analyzing EEG data, the authors aim to understand how people respond differently to images of real human faces, human-like robots, and hyper-realistic masks. The study is ambitious, focusing on isolating both sensory and cognitive neural markers associated with this unsettling phenomenon.*

Methodology: *The authors conducted two experiments with EEG, carefully analyzing neural responses at early (100–200 ms) and late (600 ms) stages. This approach is fitting, as it allows a nuanced look at both immediate and delayed neural responses, potentially linking these to initial sensory processing and later cognitive discomfort.*

Results: *The findings are compelling, suggesting neural differences at key time intervals that align with both sensory and cognitive responses. These markers could contribute to a deeper understanding of the uncanny valley.*

Contribution: *The paper makes an impactful contribution, shedding light on the neural dynamics behind human discomfort with human-like artificial entities. This is especially valuable as AI and robotics are increasingly blurring the lines between artificial and human appearance.*

❖ *General Assessment*

Overall Quality: *This is a strong, well-executed study. It combines sound methodology with a fascinating topic. While the technical elements are robust, there are parts where more clarity could be beneficial, especially in making the analysis accessible to readers from different fields.*

Significance: *The study is timely and relevant, given the current surge in AI and robotics applications. It's fascinating to consider how these findings might help designers create more "natural" interactions with digital avatars and humanoid robots, moving us closer to seamless human-machine interactions.*

Clarity and Organization: *The paper is generally well-organized, though, as with many studies in this area, simplifying certain technical terms and methods could improve the flow and broaden the audience.*

❖ *Section-by-Section Feedback*

1. Abstract

Strengths*: The abstract does a fine job summarizing the goals, methods, and outcomes of the study. It's refreshing to see a straightforward and concise overview that avoids excessive jargon.*

Improvements*: A minor point, but simplifying a few technical terms here could make it even more reader-friendly. Not everyone picking this up may be familiar with EEG-specific terms.*

2. Introduction

Strengths*: The introduction successfully grabs attention and provides a clear context for why this research matters. The uncanny valley effect is a concept that sparks curiosity, and the authors do well to build a compelling case for investigating its neural underpinnings.*

Improvements*: A few of the sentences could be streamlined to enhance readability. This isn't a major flaw, but clarity here could make it easier for non-experts to follow along.*

3. Methods

Strengths*: The experimental design is sound, and the EEG methodology is applied thoughtfully. Using multivariate pattern analysis is an excellent choice, as it allows the authors to capture the complexity of neural responses to different stimuli.*

Improvements*: Some of the statistical details, particularly around classifier accuracy, might benefit from further explanation. For readers unfamiliar with these terms, a bit more context or even a visual aid would go a long way in clarifying the approach.*

4. Results

Strengths*: The results section is clear and well-organized. The authors have done an impressive job presenting complex data in a digestible format, with Bayesian analysis adding rigor to the conclusions.*

Improvements*: Including some brief definitions for EEG terms (like P100 and N170) could enhance accessibility for readers less familiar with these concepts. Also, expanding on table and figure captions might provide extra guidance, especially for those skimming through the visuals.*

5. *Discussion*

Strengths*: The discussion provides a strong interpretation of the results. The way it links neural responses to the uncanny valley is both thought-provoking and insightful, hinting at practical applications for AI and HCI.*

Improvements*: Expanding on the potential applications of these findings would make the discussion even richer. For instance, it would be fascinating to hear more about how these insights might guide the design of AI-generated images or enhance human-robot interaction. Including potential alternative explanations, like novelty effects or cognitive dissonance, could also add depth.*

6. *Conclusion*

Strengths*: The conclusion neatly wraps up the study, summarizing the findings and potential future directions.*

Improvements*: I would suggest offering a few concrete directions for future research—maybe exploring dynamic stimuli to better simulate real-world conditions. It's a natural next step for a study of this scope.*

7. *References*

Strengths*: The references are appropriate and thorough. They cover a solid foundation of both classic and recent studies.*

Improvements*: Including some newer studies on neural responses to AI and human-like images might help connect the research to current trends in AI and digital media.*

❖ *Technological Implications*

Advancements in AI and Robotics*: The findings are highly relevant to current developments in AI and robotics. Understanding neural markers linked to discomfort with human-like artificial entities can guide the design of humanoid robots, digital avatars, and AI-generated images to avoid the uncanny valley, making interactions feel more natural and seamless.*

Improving Human-Computer Interaction (HCI): *This research offers practical insights that could benefit designers and developers across fields. By understanding how humans respond to realistic artificial faces, developers can create more engaging avatars and social robots for VR, gaming, customer service, and beyond.*

Training AI Systems: *These findings could also inform the training of AI systems, particularly generative AI, to produce human-like images or avatars that don't trigger the uncanny valley. This would be incredibly valuable for applications in media, entertainment, and even security, helping AI models produce images that are perceived positively.*

- ***Specific Comments and Technical Suggestions***

Figure Annotations*: Adding clearer labels and detailed captions for EEG components in figures could make them more intuitive.*

Technical Terms: *Defining terms like "multivariate pattern analysis" upfront would improve accessibility, making the study easier for non-experts to follow.*

Ethics Statement*: Ethical considerations are well-handled, with informed consent secured and data available through open access.*

- ***Ethical and Technical Considerations***

Ethical Standards: *The study adheres to ethical standards, with informed consent from participants and transparent data-sharing practices.*

Technical Soundness: *The methods are technically sound, though more clarity in certain terms and figure labels could make the study accessible to a broader audience.*

- ***Recommendation***

Final Recommendation: ***I strongly recommend this manuscript for publication with lightly Minor Revisions.*** *The study presents valuable insights into the neural basis of the uncanny valley effect and has significant potential to impact AI, robotics, and HCI design. Minor adjustments to clarify technical details will enhance the manuscript and make it accessible to a wider readership. This is a thought-provoking and well-conceived piece of work that deserves to be shared.*

GOOD LUCK
