# Supplementary material for: Neural correlates of the uncanny valley effect for robots and hyper-realistic masks
Source: PLoS One. 2025 Mar 18;20(3):e0311714. doi: 10.1371/journal.pone.0311714 (PMC11957730; doi:10.1371/journal.pone.0311714)
Supplement: Attachment — Submitted filename: PONE-D-24-42223-Comments.docx [file pone.0311714.s002.docx]

**Genearl comments:**

This study provides a valuable contribution to the understanding of the neural correlates of the uncanny valley effect through the use of EEG analysis on static images of androids and hyper-realistic silicone masks. The findings, such as the identification of distinct neural responses in early (100-200ms) and late (500-800ms) time windows, offer fresh insights into both bottom-up and top-down processes involved in this phenomenon. Additionally, the study demonstrates consistency across two experiments, enhancing the reliability of its conclusions.

However, there are areas that require further clarification and development to strengthen the manuscript. For example, the introduction does not fully articulate the motivation behind using static images and their advantage over dynamic stimuli in uncovering neural mechanisms. Additionally, the experimental design lacks detail regarding stimulus selection criteria and the rationale behind conditions. While EEG and behavioral data were analyzed, their integration remains insufficient, and the neural mechanisms underlying the temporal windows are not explored in depth. Furthermore, subjective ratings (e.g., uncanniness and realism) are underutilized, as their relationship with neural or behavioral data is not analyzed. Lastly, while the discussion highlights potential applications, such as improving AI-generated images, it does not provide sufficient details on how these applications might be implemented.

**Major Issues and Recommendations**

**1. Insufficient Expression of Research Motivation**

The introduction does not clearly articulate the scientific significance of the study, such as why investigating static images can complement existing research on dynamic stimuli. This may lead to ambiguity regarding the study’s uniqueness and contributions.

Recommendation: Emphasize the importance of static image research: “Although existing studies focus on dynamic stimuli, static images allow for a more precise investigation of the neural mechanisms underlying the uncanny valley effect, particularly by eliminating motion-related confounds.”

**2. Lack of Clarity in Experimental Objectives**

The description of the experimental design and objectives is too vague, failing to specify the criteria for selecting static images or the distinctions between experimental conditions.

Provide a clearer explanation of the study’s goals and design, detailing the selection process for images and the specific distinctions between conditions.

**3. Insufficient Discussion of EEG Data Analysis**

The ERP differences are only briefly discussed, without exploring the potential neural mechanisms of specific time windows (e.g., whether the 300-400ms differences are linked to cognitive conflict).

Deepen the discussion on ERP mechanisms: “The 300-400ms time window may reflect cognitive processing stages, such as determining whether a stimulus conforms to categorical expectations. Differences between human and robot conditions could indicate the unique processing advantage of human faces.”

**4. Weak Integration of Results**

There is no clear explanation of how d′ values, reaction times, and EEG data integrate to address the research question, making the relationship between methodologies unclear.

Connect different data types to answer the research question: “By integrating d′ values, reaction times, and EEG data, we explore how neural activity in specific time windows correlates with behavioral metrics like accuracy and response time.”

**5. Poor Integration of Subjective Ratings with Neural Data**

Perception ratings (e.g., uncanniness, realism) are not linked to EEG data. The connection between subjective experiences and neural signals is critical but underexplored.

Analyze the relationship between ratings and EEG data: “Higher uncanniness ratings may correspond to greater ERP differences in the late time window (600ms), indicating a link between subjective perceptions and neural responses.”

**Minor Issues and Recommendations**

**1. Weak Literature Review**

The discussion of existing research is underdeveloped, particularly regarding theoretical disagreements on the neural mechanisms (bottom-up vs. top-down processes).

Expand the literature review: “The root of the uncanny valley effect remains debated: does it arise primarily from bottom-up sensory conflicts, or from higher-level cognitive processes? Resolving this question is critical to understanding its fundamental mechanisms.”

**2. Lack of Explanation for Stimulus Presentation Time**

The rationale behind the stimulus presentation times (500ms for Experiment 1, 250ms for Experiment 2) and random blank periods is not explained.

Justify the choices: “The 500ms and 250ms durations were chosen to provide sufficient information for judgment while avoiding task fatigue. Randomized blank periods were designed to reduce carryover effects and prevent anticipatory biases.”

**3. Insufficient Analysis of Image Background Differences**

Experiment 1 used gray backgrounds, while Experiment 2 used natural backgrounds, but the potential impact of this difference is not addressed.

Discuss the impact of background differences: “While the backgrounds differed across experiments, we hypothesize that the primary task was not affected, as participants focused on the foreground stimuli.”

**4. Lack of Detail in d′ Value Calculation**

The calculation method for d′ values lacks detail, particularly for edge cases (e.g., hit rate = 1 or 0) and the rationale for capping infinite values.

Provide detailed explanations: “d′ values were capped at 5 to prevent outliers from skewing the results, following established conventions in signal detection theory.”

**5. Insufficient Interpretation of Figures**

Figures (e.g., 2 and 3) are not adequately interpreted, leaving the reader unclear about their implications.

Offer more detailed interpretations: “Figure 2a shows that robot stimuli have significantly higher d′ values than humanoids and humans, emphasizing the role of visual salience.”

**6. Limited Context for Multivariate Pattern Analysis**

High classification accuracy for human vs. robot stimuli (72%) and lower accuracy for humanoids (62%) is presented without explaining the reasons for this disparity, such as the salience of visual features.

Explore potential causes for classification differences: “The high classification accuracy for human vs. robot stimuli may be attributed to the salient mechanical elements of robots, whereas the lower accuracy for humanoids reflects their ambiguous human-like appearance, leading to confusion.”

**7. Lack of Discussion on Cross-Experiment Consistency**

While the consistency of findings across experiments is mentioned, its implications for the uncanny valley theory are not explored.

Emphasize the theoretical significance of consistent results: “The consistency of results across humanoids and hyper-realistic silicone masks suggests that the neural characteristics of the uncanny valley effect are generalizable across stimulus categories, indicating its broad applicability.”

**8. Insufficient Analysis of Temporal Windows**

The mechanisms underlying the two temporal windows (100ms and 500-800ms) are oversimplified, with no mention of their potential links to emotion processing or visual memory.

Provide detailed hypotheses for each window:

**100ms window**: “This window likely reflects rapid processing of low-level visual features, such as edges or color contrasts, consistent with P100 and N170 components.”

**500-800ms window**: “This window may involve higher-level cognitive processes, such as evaluating emotional content or judging authenticity.”

The study makes a significant contribution by identifying neural correlates of the uncanny valley effect and providing practical implications for AI and identity detection technologies. However, by addressing the above suggestions, the authors can further enhance the clarity, depth, and impact of their work.
