# Supplementary material for: Neural correlates of the uncanny valley effect for robots and hyper-realistic masks
Source: PLoS One. 2025 Mar 18;20(3):e0311714. doi: 10.1371/journal.pone.0311714 (PMC11957730; doi:10.1371/journal.pone.0311714)
Supplement: Attachment — Submitted filename: responsetoreviewsdocx.docx [file pone.0311714.s003.docx]

Reviewer #1:

R1.1 Dear Authors,
I commend you on your fascinating study of the neural correlates of the uncanny valley effect. Your methodology is solid, and the findings provide valuable insights into human responses to artificial entities, with important implications for AI and robotics design.
Please find more detailed feedback and suggestions in the reviewer attachment. In summary, I recommend the following:
1. Simplify some technical terms in the abstract and introduction for broader accessibility.
2. Provide more context and explanation in the Methods section, especially regarding statistical analysis and EEG terms.
3. Expand figure captions and define EEG terms like P100 and N170 to aid readers’ understanding.
4. Consider exploring further applications of your findings in the Discussion and including alternative explanations.
5. In the Conclusion, suggest concrete directions for future research.
6. Enhance clarity by adding clearer labels in figures and defining technical terms earlier.
I believe your manuscript has great potential and, with these minor revisions, will be ready for publication.
Best regards,

❖ Summary of the Manuscript

Title: "Neural Correlates of the Uncanny Valley Effect for Robots and Hyper-Realistic Masks"

Objective: This paper takes on the intriguing challenge of exploring neural responses linked to the uncanny valley effect. By analyzing EEG data, the authors aim to understand how people respond differently to images of real human faces, human-like robots, and hyper-realistic masks. The study is ambitious, focusing on isolating both sensory and cognitive neural markers associated with this unsettling phenomenon.

Methodology: The authors conducted two experiments with EEG, carefully analyzing neural responses at early (100–200 ms) and late (600 ms) stages. This approach is fitting, as it allows a nuanced look at both immediate and delayed neural responses, potentially linking these to initial sensory processing and later cognitive discomfort.

Results: The findings are compelling, suggesting neural differences at key time intervals that align with both sensory and cognitive responses. These markers could contribute to a deeper understanding of the uncanny valley.

Contribution: The paper makes an impactful contribution, shedding light on the neural dynamics behind human discomfort with human-like artificial entities. This is especially valuable as AI and robotics are increasingly blurring the lines between artificial and human appearance.

❖ General Assessment

Overall Quality: This is a strong, well-executed study. It combines sound methodology with a fascinating topic. While the technical elements are robust, there are parts where more clarity could be beneficial, especially in making the analysis accessible to readers from different fields.

Significance: The study is timely and relevant, given the current surge in AI and robotics applications. It’s fascinating to consider how these findings might help designers create more “natural” interactions with digital avatars and humanoid robots, moving us closer to seamless human-machine interactions.

Clarity and Organization: The paper is generally well-organized, though, as with many studies in this area, simplifying certain technical terms and methods could improve the flow and broaden the audience.

*We thank the reviewer for this enthusiastic assessment of our paper, and address the specific comments below.*

❖ Section-by-Section Feedback

R1.2 Abstract

Strengths: The abstract does a fine job summarizing the goals, methods, and outcomes of the study. It’s refreshing to see a straightforward and concise overview that avoids excessive jargon.

Improvements: A minor point, but simplifying a few technical terms here could make it even more reader-friendly. Not everyone picking this up may be familiar with EEG-specific terms.

*The only EEG-specific technical term we could see in the abstrat was “stimulus evoked potentials”, which we now clarify as referring to electromagnetic brain responses.*

R1.3 Introduction

Strengths: The introduction successfully grabs attention and provides a clear context for why this research matters. The uncanny valley effect is a concept that sparks curiosity, and the authors do well to build a compelling case for investigating its neural underpinnings.

Improvements: A few of the sentences could be streamlined to enhance readability. This isn’t a major flaw, but clarity here could make it easier for non-experts to follow along.

*We made some minor edits to enhance clarity in the Introduction.*

R1.4 Methods

Strengths: The experimental design is sound, and the EEG methodology is applied thoughtfully. Using multivariate pattern analysis is an excellent choice, as it allows the authors to capture the complexity of neural responses to different stimuli.

Improvements: Some of the statistical details, particularly around classifier accuracy, might benefit from further explanation. For readers unfamiliar with these terms, a bit more context or even a visual aid would go a long way in clarifying the approach.

*We now explain as follows:*

*“MVPA is a statistical technique that involves training a machine learning algorithm to identify patterns in data, and then testing its accuracy at classifying unseen data; in EEG analysis above-chance classification is considered evidence of distinct patterns of neural activity between two conditions.”*

R1.5 Results

Strengths: The results section is clear and well-organized. The authors have done an impressive job presenting complex data in a digestible format, with Bayesian analysis adding rigor to the conclusions.

Improvements: Including some brief definitions for EEG terms (like P100 and N170) could enhance accessibility for readers less familiar with these concepts. Also, expanding on table and figure captions might provide extra guidance, especially for those skimming through the visuals.

*We now define these components in the Fig 2 caption as follows:*

*“The P100 is a positive evoked potential occuring around 100ms after onset of a visual stimulus, associated with the initial (low level) visual response; the N170 is a negative potential at 170ms that is often associated with faces; the P200 is a further positive potential linked to attention and stimulus discrimination.”*

*And in the Discussion:*

*“The P100 is typically associated with low-level visual responses, and is affected by contrast and spatial frequency content of an image. The N170 component is most often associated with faces, though is also observed for other image categories, and there is still debate about its precise function.”*

R1.6 Discussion

Strengths: The discussion provides a strong interpretation of the results. The way it links neural responses to the uncanny valley is both thought-provoking and insightful, hinting at practical applications for AI and HCI.

Improvements: Expanding on the potential applications of these findings would make the discussion even richer. For instance, it would be fascinating to hear more about how these insights might guide the design of AI-generated images or enhance human-robot interaction. Including potential alternative explanations, like novelty effects or cognitive dissonance, could also add depth.

*We are cautious of over-reaching here, and are not experts in AI algorithms. We therefore think the current Discussion is sufficient without being too speculative, and leave it to others to potentially pick up these ideas.*

R1.7 Conclusion

Strengths: The conclusion neatly wraps up the study, summarizing the findings and potential future directions.

Improvements: I would suggest offering a few concrete directions for future research—maybe exploring dynamic stimuli to better simulate real-world conditions. It’s a natural next step for a study of this scope.

*Added:*

*“Future work might extend these findings to more dynamic stimuli, and explore potential applications for improving android and avatar generation.”*

R1.8 References

Strengths: The references are appropriate and thorough. They cover a solid foundation of both classic and recent studies.

Improvements: Including some newer studies on neural responses to AI and human-like images might help connect the research to current trends in AI and digital media.

*This is a great suggestion, though this is an even smaller literature than for the uncanny valley. We found two relevant papers (one EEG, one fMRI) which are now cited.*

R1.9 ❖ Technological Implications

Advancements in AI and Robotics: The findings are highly relevant to current developments in AI and robotics. Understanding neural markers linked to discomfort with human-like artificial entities can guide the design of humanoid robots, digital avatars, and AI-generated images to avoid the uncanny valley, making interactions feel more natural and seamless.

Improving Human-Computer Interaction (HCI): This research offers practical insights that could benefit designers and developers across fields. By understanding how humans respond to realistic artificial faces, developers can create more engaging avatars and social robots for VR, gaming, customer service, and beyond.

Training AI Systems: These findings could also inform the training of AI systems, particularly generative AI, to produce human-like images or avatars that don’t trigger the uncanny valley. This would be incredibly valuable for applications in media, entertainment, and even security, helping AI models produce images that are perceived positively.

*Indeed, we agree and have mentioned these points in the Discussion.*

R1.10 ❖ Specific Comments and Technical Suggestions

Figure Annotations: Adding clearer labels and detailed captions for EEG components in figures could make them more intuitive.

Technical Terms: Defining terms like “multivariate pattern analysis” upfront would improve accessibility, making the study easier for non-experts to follow.

Ethics Statement: Ethical considerations are well-handled, with informed consent secured and data available through open access.

*We now explain MVPA in more detail (see R1.4 above) and detail the EEG components in the Fig 2 caption (see R1.5).*

R1.11 ❖ Ethical and Technical Considerations

Ethical Standards: The study adheres to ethical standards, with informed consent from participants and transparent data-sharing practices.

Technical Soundness: The methods are technically sound, though more clarity in certain terms and figure labels could make the study accessible to a broader audience.

*We have clarified some specialist terms throughout.*

R1.12 ❖ Recommendation

Final Recommendation: I strongly recommend this manuscript for publication with lightly Minor Revisions. The study presents valuable insights into the neural basis of the uncanny valley effect and has significant potential to impact AI, robotics, and HCI design. Minor adjustments to clarify technical details will enhance the manuscript and make it accessible to a wider readership. This is a thought-provoking and well-conceived piece of work that deserves to be shared.

*Thanks again for this supportive and helpful assessment. We have addressed the comments as described above.*

Reviewer #2: The authors presented an interesting work on detecting the uncanny valley effect for robots and human-like subjects. The statistical analysis and results are well presented; however, the following changes can further improve the manuscript.

R2.1 The motivation and application of the study should also be added to the introduction section.

*We now specify that:*

*“Our aim was to further investigate neural correlates of the uncanny valley effect, with the expectation that increased understanding will aid efforts to generate more convincingly human robots and avatars in the future.”*

R2.2 The literature survey for similar works is short and needs improvement.

*There are relatively few studies on neural responses to the uncanny valley, and we think that our existing review gives a good overview of the existing literature.*

R2.3 An image of the experimentation procedure should be added.

*We provide example images of stimuli in Figure 1. Since the paradigm involved simply showing these images on a computer screen, we cannot see how a diagram of this would be useful.*

Reviewer 3: Genearl comments:

R3.1 This study provides a valuable contribution to the understanding of the neural correlates of the uncanny valley effect through the use of EEG analysis on static images of androids and hyper-realistic silicone masks. The findings, such as the identification of distinct neural responses in early (100-200ms) and late (500-800ms) time windows, offer fresh insights into both bottom-up and top-down processes involved in this phenomenon. Additionally, the study demonstrates consistency across two experiments, enhancing the reliability of its conclusions.

However, there are areas that require further clarification and development to strengthen the manuscript. For example, the introduction does not fully articulate the motivation behind using static images and their advantage over dynamic stimuli in uncovering neural mechanisms. Additionally, the experimental design lacks detail regarding stimulus selection criteria and the rationale behind conditions. While EEG and behavioral data were analyzed, their integration remains insufficient, and the neural mechanisms underlying the temporal windows are not explored in depth. Furthermore, subjective ratings (e.g., uncanniness and realism) are underutilized, as their relationship with neural or behavioral data is not analyzed. Lastly, while the discussion highlights potential applications, such as improving AI-generated images, it does not provide sufficient details on how these applications might be implemented.

*We thank the reviewer for their constructive comments and suggestions, which we address below.*

Major Issues and Recommendations

R3.2 Insufficient Expression of Research Motivation

The introduction does not clearly articulate the scientific significance of the study, such as why investigating static images can complement existing research on dynamic stimuli. This may lead to ambiguity regarding the study’s uniqueness and contributions.

Recommendation: Emphasize the importance of static image research: “Although existing studies focus on dynamic stimuli, static images allow for a more precise investigation of the neural mechanisms underlying the uncanny valley effect, particularly by eliminating motion-related confounds.”

*Now added.*

R3.3 Lack of Clarity in Experimental Objectives

The description of the experimental design and objectives is too vague, failing to specify the criteria for selecting static images or the distinctions between experimental conditions.

Provide a clearer explanation of the study’s goals and design, detailing the selection process for images and the specific distinctions between conditions.

*We now explicitly state our objectives in the Introduction:*

*“Our aim was to further investigate neural correlates of the uncanny valley effect, with the expectation that increased understanding will aid efforts to generate more convincingly human robots and avatars in the future.”*

R3.4 Insufficient Discussion of EEG Data Analysis

The ERP differences are only briefly discussed, without exploring the potential neural mechanisms of specific time windows (e.g., whether the 300-400ms differences are linked to cognitive conflict).

Deepen the discussion on ERP mechanisms: “The 300-400ms time window may reflect cognitive processing stages, such as determining whether a stimulus conforms to categorical expectations. Differences between human and robot conditions could indicate the unique processing advantage of human faces.”

*We now say in the Discussion:*

*“ERP components in later time windows have also been studied in previous work, and may reflect cognitive processing stages, such as determining whether a stimulus conforms to categorical expectations.”*

R3.5 Weak Integration of Results

There is no clear explanation of how d′ values, reaction times, and EEG data integrate to address the research question, making the relationship between methodologies unclear.

Connect different data types to answer the research question: “By integrating d′ values, reaction times, and EEG data, we explore how neural activity in specific time windows correlates with behavioral metrics like accuracy and response time.”

*We did consider doing an explicit correlational analysis along these lines, however our experiments were not powered with this in mind, and so we do not feel any conclusions would be robust. Therefore we have not included this in the manuscript.*

R3.6 Poor Integration of Subjective Ratings with Neural Data

Perception ratings (e.g., uncanniness, realism) are not linked to EEG data. The connection between subjective experiences and neural signals is critical but underexplored.

Analyze the relationship between ratings and EEG data: “Higher uncanniness ratings may correspond to greater ERP differences in the late time window (600ms), indicating a link between subjective perceptions and neural responses.”

*See our response to the previous point.*

Minor Issues and Recommendations

R3.7 Weak Literature Review

The discussion of existing research is underdeveloped, particularly regarding theoretical disagreements on the neural mechanisms (bottom-up vs. top-down processes).

Expand the literature review: “The root of the uncanny valley effect remains debated: does it arise primarily from bottom-up sensory conflicts, or from higher-level cognitive processes? Resolving this question is critical to understanding its fundamental mechanisms.”

*Added.*

R3.8 Lack of Explanation for Stimulus Presentation Time

The rationale behind the stimulus presentation times (500ms for Experiment 1, 250ms for Experiment 2) and random blank periods is not explained.

Justify the choices: “The 500ms and 250ms durations were chosen to provide sufficient information for judgment while avoiding task fatigue. Randomized blank periods were designed to reduce carryover effects and prevent anticipatory biases.”

*Added.*

R3.9 Insufficient Analysis of Image Background Differences

Experiment 1 used gray backgrounds, while Experiment 2 used natural backgrounds, but the potential impact of this difference is not addressed.

Discuss the impact of background differences: “While the backgrounds differed across experiments, we hypothesize that the primary task was not affected, as participants focused on the foreground stimuli.”

*Added in the Methods section.*

R3.10 Lack of Detail in d′ Value Calculation

The calculation method for d′ values lacks detail, particularly for edge cases (e.g., hit rate = 1 or 0) and the rationale for capping infinite values.

Provide detailed explanations: “d′ values were capped at 5 to prevent outliers from skewing the results, following established conventions in signal detection theory.”

*Now explained in the Data analysis section.*

R3.11 Insufficient Interpretation of Figures

Figures (e.g., 2 and 3) are not adequately interpreted, leaving the reader unclear about their implications.

Offer more detailed interpretations: “Figure 2a shows that robot stimuli have significantly higher d′ values than humanoids and humans, emphasizing the role of visual salience.”

*We added:*

*“The higher d' values for robots (Figure 2a) could indicate that these stimuli are more visually salient.”*

R3.12 Limited Context for Multivariate Pattern Analysis

High classification accuracy for human vs. robot stimuli (72%) and lower accuracy for humanoids (62%) is presented without explaining the reasons for this disparity, such as the salience of visual features.

Explore potential causes for classification differences: “The high classification accuracy for human vs. robot stimuli may be attributed to the salient mechanical elements of robots, whereas the lower accuracy for humanoids reflects their ambiguous human-like appearance, leading to confusion.”

*Added.*

R3.13 Lack of Discussion on Cross-Experiment Consistency

While the consistency of findings across experiments is mentioned, its implications for the uncanny valley theory are not explored.

Emphasize the theoretical significance of consistent results: “The consistency of results across humanoids and hyper-realistic silicone masks suggests that the neural characteristics of the uncanny valley effect are generalizable across stimulus categories, indicating its broad applicability.”

*The final sentence of the first paragraph in the Discussion now reads:*

*“The similarity in results across our two experiments is striking and constitutes an internal conceptual replication of our main findings, suggesting that the neural characteristics of the uncanny valley effect are generalizable across stimulus categories.”*

R3.14 Insufficient Analysis of Temporal Windows

The mechanisms underlying the two temporal windows (100ms and 500-800ms) are oversimplified, with no mention of their potential links to emotion processing or visual memory.

Provide detailed hypotheses for each window:

100ms window: “This window likely reflects rapid processing of low-level visual features, such as edges or color contrasts, consistent with P100 and N170 components.”

500-800ms window: “This window may involve higher-level cognitive processes, such as evaluating emotional content or judging authenticity.”

*Added in the Results section.*

R3.15 The study makes a significant contribution by identifying neural correlates of the uncanny valley effect and providing practical implications for AI and identity detection technologies. However, by addressing the above suggestions, the authors can further enhance the clarity, depth, and impact of their work.

*We thank the reviewer again for their comments and suggestions, which have been addressed as outlined above.*
